# Prosodic signatures of ASD severity and developmental delay in preschoolers

**DOI:** 10.1038/s41746-023-00845-4

**Published:** 2023-05-29

**Authors:** Michel Godel, François Robain, Fiona Journal, Nada Kojovic, Kenza Latrèche, Ghislaine Dehaene-Lambertz, Marie Schaer

**Affiliations:** 1 Department of Psychiatry, University of Geneva School of Medicine, Geneva, Switzerland; 2 Cognitive Neuroimaging Unit, CNRS ERL 9003, INSERM U992, CEA, Université Paris-Saclay, NeuroSpin Center, Gif/Yvette, France

**Keywords:** Autism spectrum disorders, Prognostic markers

## Abstract

Atypical prosody in speech production is a core feature of Autism Spectrum Disorder (ASD) that can impact everyday life communication. Because the ability to modulate prosody develops around the age of speech acquisition, it might be affected by ASD symptoms and developmental delays that emerge at the same period. Here, we investigated the existence of a prosodic signature of developmental level and ASD symptom severity in a sample of 74 autistic preschoolers. We first developed an original diarization pipeline to extract preschoolers’ vocalizations from recordings of naturalistic social interactions. Using this novel approach, we then found a robust voice quality signature of ASD developmental difficulties in preschoolers. Furthermore, some prosodic measures were associated with one year later outcome in participants who had not acquired speech yet. Altogether, our results highlight the potential benefits of automatized diarization algorithms and prosodic metrics for digital phenotyping in psychiatry, helping clinicians establish early diagnosis and prognosis.

## Introduction

Prosody refers to the suprasegmental aspects of speech that can be modulated to enhance meaning and emotions^1^. The prosody of an utterance is constituted by its intonation, i.e., whether the pitch is globally high or low and the dynamic contour it follows (rising, falling or more complex patterns). Prosody also refers to the rhythm (fast, slow, jittery), loudness (loud or quiet) and voice quality (hoarse, nasal, creaky) of a vocalization. Prosody plays a major role in verbal communication. For instance, it conveys critical information about the grammatical structure of the sentence, such as pauses indicating the boundary between phrases^2^, the pragmatic context (such as sarcasm^3^) and the speaker’s affective state^4,5^. Thus, impaired prosody production observed in some neurodevelopmental disorders can signal different levels of difficulties in communication and social interaction^6–8^. In the present study, we explored prosody in early Autism Spectrum Disorder (ASD). We collected acoustic measures of prosody in a sample of preschoolers with ASD, an age that is crucial for the acquisition of speech and its prosodic aspects. Our aim was to explore how prosody relates to developmental delays and ASD symptoms within a young autistic sample displaying heterogeneous clinical profiles.

ASD is defined by difficulties in social interactions and communication associated with repetitive behaviors and/or restricted interests^9^. In his very first clinical report of the disorder, Leo Kanner depicted the voice of 7 y.o. Elaine C. as “unmodulated” and “hoarse”^10^. Since Kanner’s seminal report, clinicians have consistently described specific prosodic features in individuals with ASD^11,12^. Common clinical descriptions include atypical rhythm (inappropriate stressing), intonation (unusual high pitch, ‘singsong’ voice or monotonous intonation), voice quality (squeaky or hoarse voice) and loudness (socially inappropriate shouting or whispering). Nowadays, the Autism Diagnostic Observation Schedule (ADOS) which is the most commonly used diagnostic tool for ASD considers prosody as one of the clinically significant signs for diagnostic decision^13^.

Over the past two decades, acoustic analyses have been undertaken to objectively quantify the prosodic specificities long-time described in ASD. The most robust differences found between ASD and their typically developing (TD) peers concern the domain of intonation^14–16^. Autistic children and adults exhibit larger pitch range^17–21^ and higher pitch^19–21^ compared to TD. Regarding prosodic rhythm, one study has found that school-aged children with ASD (*n* = 41) exhibit slower speech rate when asked to name pictures compared to TD (*n* = 42)^17^. When exploring voice quality, Bone et al. (2014) have found a correlation between ASD symptoms and higher jitter (i.e., the fluctuation of the fundamental frequency between cycles of glottis opening/closure) as well as higher jitter variability when analyzing short excerpts of naturalistic social interactions in a group of 28 school-aged children with ASD^22^. Moreover, a recent study where 33 children (13 with ASD and 20 TD) were asked to read words has reported higher vocalization strength in ASD^20^.

To sum up, many acoustic measures of prosody have been reported to differ between ASD and TD. Nonetheless, few studies have explored prosody within the specific population of autistic preschoolers and its associations with developmental phenotypes. Two recent independent studies have reported a correlation between ASD symptom severity and higher pitch in preschoolers with ASD^23,24^. The lack of prosody studies on this population has been recently identified as a research gap in ASD^15,16,25,26^. First reliable ASD diagnosis are generally set during preschool age^27^, hence better characterization of the relations between prosodic and behavioral measures at this age could help develop new automated diagnostic/prognostic classification tools^25^. Helping clinical appreciation using algorithms and individual digital information such as vocal recording is the scope of digital psychiatry^28,29^. This emerging research area has already shown great potential for ASD and other heterogeneous disorders^30,31^.

One reason for the prosody research gap in autistic preschoolers is that vocal recordings at this age can be highly cumbersome to preprocess. It can be difficult to ask preschoolers with ASD to name pictures, tell a story or read a text in front of a microphone as it is commonly done with older populations^18,20,32^. Hence, exploring prosody in autistic preschoolers generally requires the collection of spontaneous vocalizations, such as those occurring during a naturalistic social interaction with an adult. In this perspective, authors have often recorded preschoolers during the ADOS which offers a standardized setting for social interaction^33,34^. Although social interaction recordings provide ecologically valid prosody compared to reading texts or naming pictures^12,35,36^, they contain noise and adults’ voices requiring to be manually removed before analyzing participants’ prosody. A promising alternative to this time-consuming manual preprocessing relies on the application of diarization algorithms, i.e., techniques that automatically determine *who spoke when* in audio recordings^37^. Commonly used diarization methods are based on acoustic energy information to infer the spatial position of each speaker, e.g., using two simultaneous recordings^38^ or a single microphone held close to the participant’s mouth^23,24,39,40^. However, multi-channel recordings or microphones fixed close to the mouth are not commonly used when collecting social interaction recordings in cohorts of autistic preschoolers. Yet, if diarization algorithms existed and worked on single-channel ADOS recording, they could be readily used a posteriori on any previous recording to alleviate the amount of time spent on extracting the participants’ vocalizations. In the scope of digital psychiatry, diarization algorithms allowing a large collection of vocal data in preschoolers with ASD could help train algorithms identifying early ASD diagnostic/prognostic patterns^23,24^.

Here, we explored how spontaneous prosody production relates to developmental delay and ASD symptom severity phenotypes in autistic preschoolers. With the aim of reducing manual pre-processing time and in view of the lack of existing methods, we present a novel diarization pipeline to automatically extract children vocalizations from single-channel ADOS recordings. We applied our original diarization method on a sample of 74 preschoolers with ASD aged 1.5–6 y.o. displaying heterogeneous levels of development and ASD symptoms. We extracted prosodic parameters related to intonation, loudness, rhythm and voice quality using the Geneva Minimalistic Acoustic Parameter Set (GeMAPS)^41^ and examined the multivariate relations governing prosody, developmental measures and ASD symptom severity using partial least square correlations (PLSC). Finally, we performed longitudinal analyses on participants who had not developed speech yet to explore whether some prosodic features might predict the prognosis 1 year later.

## Results

### Prosodic patterns in preschoolers with and without phrase speech

**Fig. 1:**
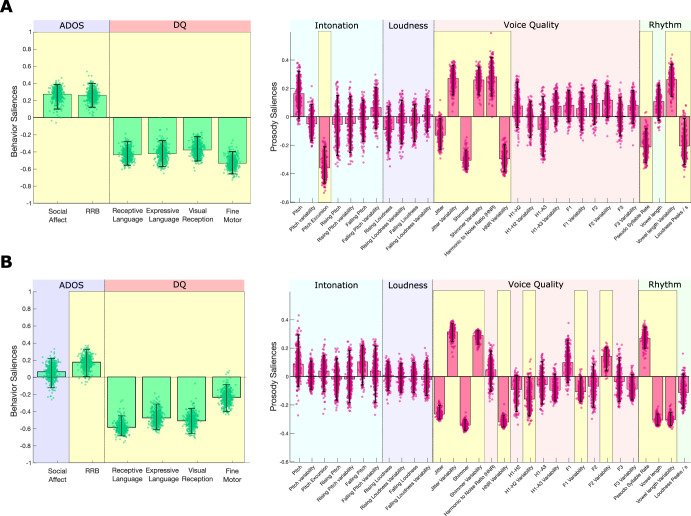
Prosody in autistic preschoolers with and without phrase speech. **A** Partial Least Square Correlation (PLSC) in *preschoolers with ASD without phrase speech* (PreSp, *n* = 42). PLSC explored the association between behavioral (ADOS CSS in blue and DQ in pink) and prosodic measures relating to intonation (blue), loudness (purple), quality of voice (pink) and rhythm (green). Behavioral and prosodic saliencies of the first principal component are displayed. Error bars represent 5–95th percentiles of 500 bootstrap samples, and robust results are highlighted in yellow. **B** PLSC in *autistic preschoolers with phrase speech* (Sp, *n* = 32). ADOS Autism Diagnostic Observation Schedule, CSS Calibrated Severity Score, DQ Developmental Quotient, RRB Repetitive and Restricted Behaviors.

We investigated prosody in two independent groups, each of them displaying relatively homogeneous types of vocal productions, namely a speech group (Sp, *n* = 32) that includes children assessed with the ADOS Module 2 or 3, and a Prespeech group (PreSp, *n* = 42) comprising children assessed with the ADOS Toddler Module or Module 1. Modules 2 and 3 are administered to participants who use spontaneous and meaningful three-word utterances that occasionally include a verb (i.e., “phrase speech” as defined by the ADOS). Toddler Module and Module 1 were administered to participants who have not reached this stage of language production. We explored the associations between prosodic measures and behavioral phenotype within each group separately by applying Partial Least Square Correlation (PLSC, see Methods). We used developmental quotients (DQ) and ASD calibrated severity scores (CSS) from the ADOS as behavioral variables and analyzed their multivariate correlation patterns with prosodic measures related to intonation, loudness, voice quality and rhythm. Age, gender and the type of microphone used were regressed out within each group. PLSC resulted in a single significant correlation component in PreSp (*p* = 0.028, *r* = 0.65, Fig. 1A) and another one in Sp (*p* = 0.004, *r* = 0.63, Fig. 1B). In both PreSp and Sp groups, prosodic measures were strongly associated with DQ and, to a lesser extent (i.e., lower Bootstrap Ratios or BSR), to ASD symptom severity.

In PreSp, lower DQ and higher ASD symptom severity were associated with intonation (flatter and higher pitch), voice quality and rhythmic (slower pseudo syllable rate) metrics (Fig. 1A). More specifically, decreased pitch excursion (i.e., local variations in pitch measured in semitones per second, BSR = −6.227) and higher pitch (BSR = 2.276) were associated with higher ASD symptom severity and lower DQ (all BSR are reported in Supplementary Table). Our results extend previously published positive correlation between pitch and ASD symptom severity in preschoolers with heterogeneous levels of language production^23,24^. In terms of PreSp rhythmic metrics, we found an expected association between lower DQ with higher ASD symptom severity and indicators of weaker use of words and/or babbling (decreased pseudo syllabic rate, BSR = −3.717, and increased vowel length variability, BSR = 5.281). Our finding on decreased pseudo syllabic rate in PreSp is in line with the results of Wetherby (2007) showing reduced consonants inventory in toddlers with ASD and lower DQ. Finally, we found that in PreSp, DQ and ASD symptoms were associated with various voice quality measures. More specifically, lower DQs with higher ASD symptom severity were associated with lower harmonic to noise ratio (HNR) variability (BSR = 4.405) as well as with jitter and shimmer that were globally decreased (BSR for jitter = −2.478 and −7.758 for shimmer) and more variable over time (BSR = 5.798 for jitter variability and 5.725 for shimmer variability). An alteration of the same measures has been reported in preschoolers^16,24^ and older individuals^22,42^ with ASD compared to TD.

In the Sp sample, lower DQ and higher Restricted and Repetitive Behavior (RRB) were linked to voice quality and rhythmic (faster pseudo syllable rate) measures (Fig. 1B). On the whole, prosody was associated with Restricted and Repetitive Behavior (RRB) (BSR = 2.353) but not with Social Affect (SA, BSR = 0.771), a contrast that has been previously reported by Eni M et al. (2020) in a similar population^24^. Unlike in PreSp, we found no association between intonation metrics and behavior in the Sp group, as previously reported by Diehl et al. (2009) and Nadig and Shaw (2012)^18,43^. In terms of rhythmic measures, Sp participants with lower DQ showed increased pseudo syllabic rate (BSR = 7.251) with less variable vowel length (BSR = −15.361), i.e., a reverse pattern compared to our PreSp results (Fig. 1A). Finally, the pattern of association between voice quality parameters and behavior in Sp was highly similar to the one we reported in PreSp, namely lower DQ and higher ASD symptoms associated with lower HNR variability (BSR = −17.016), decreased jitter (BSR = −12.139) and shimmer (BSR = −17.494), more variable jitter (BSR = 9.852) and shimmer (BSR = 13.072). In addition, Sp participants showed an association between developmental difficulties and higher variability of second formant (BSR = 3.890), a result that has been previously reported in older autistic children^20^.

Furthermore, we found no association between loudness measures and behavior in either PreSp or Sp (BSR < 2.3 for all loudness measures in both groups PLSC). This result is in line with many null findings of previous studies exploring loudness metrics in ASD (see Fusaroli et al., 2017 for a systematic review). Nonetheless, loudness measures highly depend on the standardization of the recording setting, i.e., controlling for the distance between microphone and participant during the evaluation, which was not the case in our protocol. Thus, this null result might reflect an insufficiently standardized recording setting.

### Language production stage influences some prosodic measures, age does not

**Fig. 2:**
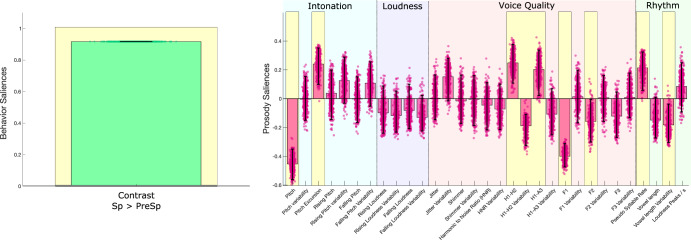
Association between prosody and language production stage. Significant correlation component of Partial Least Square Correlation (PLSC) in 74 preschoolers with ASD is displayed. PLSC explored the association between behavioral contrast (Sp versus PreSp) and prosodic measures relating to intonation (blue), loudness (purple), quality of voice (pink) and rhythm (green). Behavioral contrast and prosodic saliencies of the first principal component are displayed. Error bars represent 5–95th percentiles of 500 bootstrap samples, and robust results are highlighted in yellow. PreSp: without phrase speech; Sp: with phrase speech.

We first explored the prosody-developmental associations within each group to avoid a potential confounding effect between the level of vocal production (ability to produce utterances with more than two words or not) and prosodic metrics. In a second step, we applied PLSC on the whole sample (*n* = 74) using speech production level (PreSp or Sp) as the single behavioral contrast (See Methods) to explore the possible confounding effect of language production stage on prosodic measures. Age, gender and the type of microphone used were regressed out within each group. PLSC resulted in a single significant correlation component (*p* < 0.001, *r* = 0.56, Fig. 2).

When using language production stage as the behavioral contrast (i.e., Sp versus PreSp), we found differences between PreSp and Sp groups in intonation, voice quality and rhythmic measures (Fig. 2). Higher production abilities (i.e., phrase speech) were associated with lower pitch (BSR = −9.556), increased pitch excursion (BSR = 4.187), increased pseudo syllable rate (BSR = 3.598) and decreased variability of vowel length (BSR = −2.656). In PreSp, a higher DQ was associated with increased pitch excursion and pseudo syllabic rate (Fig. 1A). The association we found between those measures and the stage of language production (Fig. 2) suggests that associations between behavior, rhythm and intonation in PreSp (Fig. 1A) could have been partly influenced by more advanced stages of language production in participants with higher DQ. In contrast, associations between behavior and rhythmic prosody in Sp (Fig. 1B) did not follow the same directionality as the association between prosody and language production stage (Fig. 2). This contrasting finding suggests that faster syllabic rates in Sp participants with lower DQ (Fig. 1B) were driven by prosody itself and not by decreased complexity of language productions. Finally, the voice quality alterations that were shared by both groups (jitter, shimmer and HNR, Fig. 1A and B) were not linked to language stage (Fig. 2).

We also wanted to rule out a potential confounding effect of age on prosodic measures, even though age had been regressed out from all analyses. To do so, we ran a PLSC on the whole sample using prosodic metrics with age as the single behavioral variable. Gender, the type of microphone used, and the language production stage (PreSp or Sp) were regressed out. We found no significant correlation component (the most significant component had a *p* value equal to 0.200). This suggests that in autistic preschoolers, age has a minimal influence on prosodic metrics compared to DQ, ASD symptoms and the language production stage.

### Prosody as prognostic marker

**Fig. 3:**
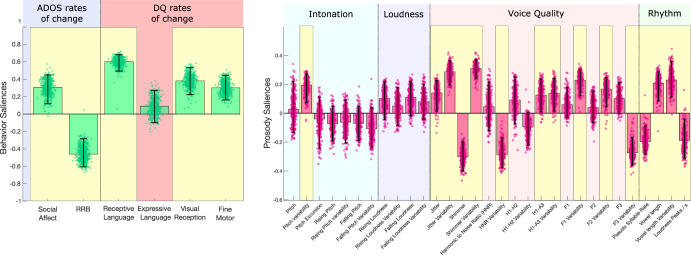
Associations between prosody and subsequent behavioral rates of change. PLSC in a subsample (*n* = 32) of preschoolers without phrase speech (PreSp). PLSC explored the association between the prosodic measures at the first visit and the behavioral rates of change (SPC) in ADOS CSS (in blue) and DQ (in pink) during the following year. Prosodic measures comprised intonation (blue), loudness (purple), quality of voice (pink) and rhythm (green). Behavioral and prosodic saliencies of the first principal component are displayed. Error bars represent 5–95th percentiles of 500 bootstrap samples, and robust results are highlighted in yellow. ADOS Autism Diagnostic Observation Schedule, CSS Calibrated Severity Score, DQ Developmental Quotient, RRB Repetitive and Restricted Behaviors, SPC Symmetrized Percent Change.

Finally, in the PreSp group for which ADOS and DQ had been collected 1 year later (*n* = 32), we analyzed the relation between prosodic parameters on the first visit and behavioral rates of change during the following year. Age, gender and the type of microphone used were regressed out. In this subsample, the PLSC analysis revealed a single significant correlation component (*p* = 0.003, *r* = 0.62, Fig. 3).

In PreSp, some measures of intonation, voice quality and rhythm were associated with better outcome in DQ and RRB (Fig. 3). We found a specific pattern of prosody associated with a better developmental outcome 1 year later, namely the improvement in DQ (except for expressive language, BSR = 1.148) and a decrease in the RRB symptom severity (BSR = −5.429). A pitch that was more variable throughout the recording was correlated with better developmental and RRB outcome (pitch variability, BSR = 4.245). Moreover, better DQ and RRB prognosis in PreSp were linked to less dense use of syllables, i.e., a rhythmic pattern that is similar to the one we found in Sp with higher DQs (See Fig. 1B).

## Discussion

Here, we applied an original diarization pipeline on a large sample of autistic preschoolers engaged in a semi-structured naturalistic social interaction with an adult male examiner (*n* = 74). Our algorithm showed robust performance in automatically classifying recorded children voices (87.5% sensitivity on a validation sample, see Supplementary Methods), thus drastically decreasing the amount of time required to manually extract preschoolers’ vocal productions. In our sample, intonation modulation, voice quality and rhythm were related to developmental delay and ASD symptom severity at the time of the recording, as well as the following year. Firstly, we discuss in this section how multivariate approaches in heterogeneous autistic samples provide a fine-grained understanding of prosodic development in early ASD, especially in the rhythmic domain. Secondly, we discuss in more detail our findings in the participants without speech (PreSp) and how they could fit with existing models of autistic prosody, with a special focus on fine motor and intonation results. Thirdly, we discuss the potential contribution of our work to the field of digital psychiatry, given the performances of our diarization method as well as the ASD prosodic markers of prognosis that we found.

Most prosody studies to date have focused on relatively homogeneous ASD populations with preserved cognitive and language abilities, an issue raised by different authors^15,16,26^. For instance, previous studies exploring rhythmic prosody have reported no difference between ASD without language or cognitive impairment and their TD peers^43,44^. However, by restricting analyses to autistic children with higher level abilities, these studies might have missed protracted atypical prosody observed in more heterogeneous groups as ours. Our original diarization algorithm allowed us to collect large samples of vocal production in preschoolers that are representative of the full ASD developmental heterogeneity. In contrast to Grossman et al. (2010) and Nadig and Shaw (2012), both our PreSp and our Sp groups exhibit alterations of rhythmic prosody in relation to developmental level and ASD symptom severity. Not only did we discover a link between rhythmic prosody and developmental measures in early ASD, but we also highlighted a fine-grained dissociation in the rhythmic prosody-development association according to the language production stage (Fig. 1). Namely, PreSp participants with lower DQ produced syllables with less density and more variable length (i.e., slow and unstable syllables), whereas Sp participants with the same developmental profile (lower DQ) showed the exact opposite prosodic pattern, i.e., syllables that were denser and more stable in length. This rhythmic contrast between PreSp and Sp exhibiting lower DQ suggests that once speech production is established in ASD (i.e., Sp), higher developmental abilities allow more modulated rhythmic control, thus resulting in slower syllabic rate with more variable vowel length. Conversely, before the emergence of speech (i.e., PreSp), higher DQ might be associated with more frequent babbling and/or use of single words, thus resulting in increased pseudo syllable rate measure^45,46^. We also found that pseudo syllable rate globally increased with speech acquisition (Fig. 2), probably because of the high temporal density of syllabic production in phrase speech. Taking everything into consideration, these results suggest that prosodic rhythmic measures in ASD are greatly affected by language production stage, as phrase speech acquisition was associated with globally denser syllable use and a full reversal of DQ-rhythmic correlations. This conclusion converges with the claim of Vargason et al. (2020) insisting on the importance of using multivariate statistical approaches including many behavioral variables on populations expressing the full ASD spectrum when exploring potential physiological markers of ASD^47^.

Our results shed some light on the early mechanistic underpinnings of prosody in autistic children, especially in the period before speech emergence. Given our results, it appears that fine motor abilities and intonation both play an important role during PreSp stages of ASD. In Presp (Fig. 1A), fine motor (i.e., motor planning and control of hands^48^) was the developmental domain showing the highest salience in the multivariate correlation component governing prosody-behavior associations. This finding fits well with previous results suggesting that atypical prosody in ASD could be partly caused by broader fine motor difficulties^49–51^. According to this view, impaired prosody regulation would result from a poor control over the complex motor units implicated in language production. Around 100 muscles should be perfectly coordinated at a fast pace for an adequate modulation of vocal production^52^. Thus, any deficit in cerebral and/or cerebellar motor areas underlying fine motor might impact the coordination of agonist and antagonist muscles required for both adequate vocal modulation and hands motor control and planning. This complex entanglement between vocal and hand motor control is particularly relevant in ASD in which fine motor abilities are known to greatly affect expressive language development, especially before the emergence of speech^53–55^. A recent study has found a causal relationship between fine motor delay and poor language intelligibility in a subgroup of autistic children without speech^56^. Our results in PreSp suggest that intelligibility in this population could be affected by fine motor delay through its effects on prosody. Prosody, which is a subdomain of expressive language, could therefore be a sensitive measure of fine motor delays that could be used in digital phenotyping approaches, a topic that we will discuss later in this section. Moreover, we observed that in PreSp, decreased intonation modulation (captured by pitch excursion and pitch variability measures) was related to concurrent as well as to later developmental delay severity (Figs. 1A and 3). Such associations were no longer found once phrase speech was acquired (Fig. 1B), thus suggesting a mechanism that is specific to PreSp. More specifically, locally increased modulation (i.e., higher pitch excursion) was associated with higher DQs and less ASD symptoms, although a potential confounding effect of language production stage cannot be ruled out (Fig. 2). Furthermore, higher pitch variability was associated with higher DQ and less RRB 1 year later (Fig. 3). The altered intonation patterns in PreSp participants might reflect the persistence of prosodic features that have been reported in infants who later develop ASD^57,58^. Moreover, early intonation modulation in TD constitutes a marker of later speech production abilities^59,60^ that can be used to estimate language outcome^61,62^. Our findings thus extend these TD characteristics to the ASD population in which the PreSp ability to modulate intonation could represent a marker of concurrent and later developmental difficulties and ASD symptom severity. We would also like to highlight some limitations of the interpretation of our PreSp results. As a first limitation, we didn’t label vocal productions of PreSp according to their qualitative type - e.g., screams, “*jargon*”, laughs or syllable reduplications that are frequent in PreSp ASD. Hence, we cannot fully grasp the relative contribution of each specific type of vocal production to the prosodic measures. For instance, one could speculate that increased pseudo syllable rate in a PreSp recording segment could be a marker of the preverbal RRB consisting in self-stimulating reduplication of syllables. Thus, applying an approach similar to ours that would include vocal qualitative typology, one would bring a more fine-grained interpretation of our results. The second limitation relies on the absence of a control TD sample, thus precluding the estimation of how specific to ASD the relation we found between intonation modulation and behavioral trajectories is. Oller et al., (2010) have showed for instance that prosodic rhythm and voice quality correlate with age in TD, but not in ASD, suggesting that TD and ASD could experience distinct patterns of interactions between development and prosody^39^. It is worth noting that since we did not have a control group of individuals with non-autistic developmental delays, we cannot rule out the possibility that the associations we observed between prosody and developmental delays are not unique to ASD. Future studies including preverbal non-autistic children with delays in fine motor skills are crucial to further explore the relation between motor control and atypical prosody. But despite these limitations, our results in PreSp converge with the hypothesis of an association between fine motor control and prosody acquisition in ASD, even suggesting that this association could be particularly relevant in the early stage of speech acquisition. Our findings also suggest that a poorer intonation control during the PreSp stage could serve as a marker of concurrent and later developmental difficulties, an association that has already been demonstrated in TD populations.

Finally, our original diarization pipeline as well as the associations we found between prosodic and behavioral phenotypes could inspire concrete applications in digital phenotyping approaches^28,29^. Up to now, digital phenotyping in ASD has mostly focused on computer vision analysis, showing great potential in efficiently stratifying various clinical dimensions of early ASD^63,64^. In contrast, attempts to obtain digital phenotypes from preschoolers with ASD using vocal metrics have remained scarce, partly because of the lack of effective diarization methods applicable on common recording settings^23,24^. Our original diarization approach could thus facilitate the development of vocal digital phenotyping tools. In this study, we restricted our diarization pipeline to recordings of preschoolers interacting with a male adult to maximize the performance of the classifier as a first approach. However, future development of our approach could rely on its extension to recordings including adult female examiners and/or more than two speakers. When we tested our classifier (i.e., that had been trained with male examiners) on female adults, the resulting tracks contained approximatively five times more incorrect data that would need to be manually removed (with the same amount of children voice data kept for analyses, see Methods). Alternatively, training a classifier using only adult female voices resulted in tracks with far less incorrect data to be cleaned, but at the cost of important loss of children voice data that were incorrectly classified. Finally, training a classifier with all three types of voices yielded performances that were a compromise between the two first approaches, i.e., less child voice data loss than when trained only on female adults, but more incorrectly classified adult voice compared to classifier trained only on adult males. Although our diarization approach performances were poorer when trained and/or applied using adult female voices, they would still result in far less preprocessing time compared to full manual extraction of children voice data from a recording. In addition, there are multiple strategies to improve our diarization model when applied on adult female voice. For instance, one could include more acoustic parameters in the discriminant analysis. Beyond the classic age-related parameters of fundamental frequency and formants that we used here, other measures such as the Cepstral/Spectral Index of Dysphonia or the Degree of Subharmonics have been recently identified as vocal markers of age in both males and females^65^. Alternatively, deep machine learning approaches could be used to determine without any a priori what are the best acoustic measures to include in our diarization model to classify adult female *versus* children voices. Despite this methodological limitation, we found a specific prosodic pattern associated with a better outcome 1 year later in PreSp (Fig. 3). For instance, in PreSp, some rhythmic characteristics prefiguring the specific prosody of Sp participants with higher developmental levels (e.g., slower pseudo syllable rate) were indicative of better developmental prognosis. This suggests that some prosodic features that are specific to higher DQ autistic speech might already be noticeable in pre-speech vocal productions and thus serve as prognostic markers. Studies using longitudinal designs are required to further confirm this hypothesis. Altogether, this result highlights the potential of automated vocal analyses in individualized prognostic estimation. Furthermore, we found a set of three voice quality parameters (jitter, shimmer and harmonic to noise ratio, HNR) associated with DQ and ASD symptom severity that was consistent across two independent groups displaying different levels of language production (PreSp and Sp, Fig. 1A and B). Jitter, shimmer and HNR have previously been found to be atypical in ASD^16,22,24,42^. Moreover, none of these measures were affected by participants’ language production stage (Fig. 2), further indicating that they could be used as developmental markers in ASD regardless of individuals’ expressive level. To sum up, this overall similarity between PreSp and Sp patterns advocates for a robust voice quality signature of developmental delay and ASD symptom severity that is shared across ages and levels of language production in autistic preschoolers. In the future, this set of voice quality measures could be selected a priori to develop digital phenotyping tools using acoustic data.

In conclusion, we present here a new diarization approach to extract preschoolers’ vocal productions from naturalistic interactions between a child and a male adult. Using this new method, we were able to collect a large sample of voices from autistic preschoolers with highly heterogeneous levels of ASD symptoms and developmental difficulties. We applied multivariate statistical analyses on this population that has been scarcely studied in terms of age and clinical profile. We highlighted some fine-grained patterns of associations between prosody, developmental delays, ASD symptoms and language production stage. Before the emergence of speech, intonation control and fine motor control appeared to play a major role, whereas rhythmic prosody exhibited a complex pattern of evolution that highly depended on the language production stage. Furthermore, voice quality appeared as a robust marker of developmental difficulties in ASD, regardless of the level of language expressive abilities. In the future, studies that include preschoolers with TD and/or non-autistic developmental delays are needed. Increased generalization performances of our diarization algorithm when used with female adults would widen the potential applications of our approach. Altogether, our study highlights the importance of exploring prosody at early stages of ASD within the full heterogeneity of the spectrum using multivariate strategies. It also brings new perspectives in the use of prosodic metrics in digital psychiatry, notably to assist prognostic evaluation.

## Methods

### Participants

**Fig. 4:**
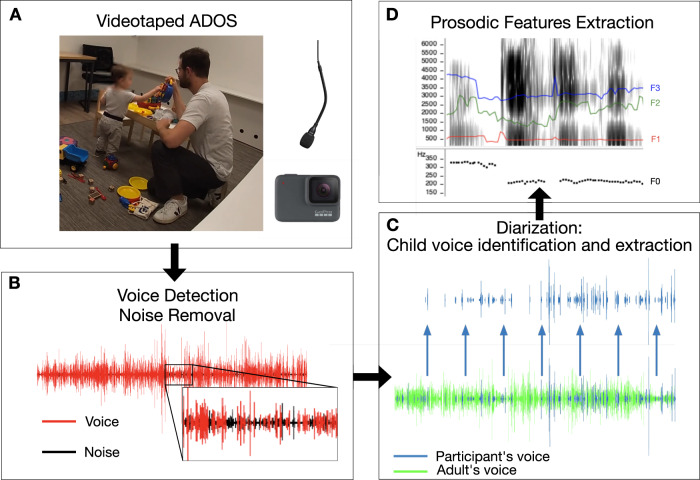
Summary of the preprocessing pipeline. **A** Recording of the ADOS administered by a male examiner with either a Shure© MX202 Microflex® overhead microphone or the GoPro© Hero7 built-in microphone. **B** After pre-emphasis filtering and denoising (not illustrated), a voice detection algorithm was applied on the complete audio track. Noise was removed and voiced segments were concatenated. **C** Then, we applied our original diarization algorithm to automatically identify, extract and concatenate the segments of children vocalizations. The resulting concatenated audio track was carefully inspected and edited by an examiner (not illustrated). **D** Ultimately, a set of prosodic parameters were extracted from the isolated children vocal productions using the GeMAP pipeline. Illustration of prosodic features (above right) was obtained using WaveSurfer.js for illustration purposes. Here, we display pitch contour (below) and formants trajectories on a spectrogram (above) of a 3 s excerpt from a single participant. Informed consent for the photograph displayed was signed by the participant’s parents. F0 relates to fundamental frequency (or pitch) and F1, F2 and F3 relate to first, second and third formants respectively. ADOS Autism Diagnosis Observation Schedule.

Participants were part of the ongoing longitudinal *Geneva Autism Cohort* study (see Franchini et al., 2018 for a description of the longitudinal design^66^). Preschoolers with either typical development (TD) or Autism Spectrum Disorder (ASD) were recruited through parent associations, clinical centers and announcements throughout the Geneva community. ASD diagnosis was confirmed by a licensed child psychiatrist (MS) using the Diagnostic and Statistical Manual of mental disorders, 5th edition^9^ criteria, combined with the Autism Diagnostic Observation Schedule (ADOS) diagnostic cut-off^13,67^. Parents of all participants gave their written and informed consent. Informed consent for the photograph displayed on Fig. 4 was signed by the participant’s parents. All signed consent forms are stored in compliance with local confidentiality laws. The research protocol was approved by the review board of the University of Geneva. In the present manuscript, we use both identity-first language (e.g., “autistic preschoolers”) and person-first language (e.g., “participant with ASD”) to reflect the diversity of semantic preferences in the ASD community^68^.

Participants assessments comprised the Mullen Scales of Early Learning (MSEL)^48^, the Psychoeducational Profile – third edition (PEP-3)^69^ and the ADOS. MSEL and PEP-3 allow the computation of individual developmental quotients (DQs)^70,71^.

The Mullen Scales of Early Learning (MSEL) is designed for children aged from birth to 68 months. The MSEL is a standardized assessment that measures the participant’s development in five distinct domains of development. The evaluated domains consist in receptive language (RL), expressive language (EL), fine motor (FM), and visual reception (VR). Gross motor (GM) is also evaluated but is not considered in the computation of the composite score that reflects the overall child’s level of development.

The Psychoeducational Profile – third edition (PEP-3) is another standardized developmental evaluation designed for children aged from 2 to 7 years. Evaluated domains include EL, RL, FM, GM, visuo-motor imitation, and cognitive verbal and preverbal (CVP).

We computed the Developmental quotient scores (DQ) for each domain of the MSEL by dividing individual developmental age by chronological age multiplied by 100^70^. Each participant’s composite DQ was computed by averaging his/her DQs in FM, VR, RL and EL. MSEL was not collected for 6 participants (4 PreSp and 2 Sp). To increase sample size, we included these 6 participants by using their DQ derived from the PEP-3. For the PEP-3 composite DQ computation, VR was replaced by CVP which is the domain showing the most overlap in tested items^71^. Compared to standard scores, DQ offers the advantage of limiting the truncation of scores from very low performing participants.

The ADOS is a standardized semi-structured evaluation that aims to recreate as much as possible a naturalistic interaction between a child and an adult^13^. The ADOS usually takes between 30 and 60 min to be administered. There are five different modules according to participant’s age and level of language. Namely, Module 2 and above are designed for individuals using “phrase speech”, defined as spontaneous and meaningful three-word utterances that occasionally include a verb. Module 1 and Toddler are administered to individuals who don’t use “phrase speech”, i.e., with no word to two-word combinations. The ADOS allows a quantification of ASD symptoms by computing a Calibrated Severity Score (CSS) in Social Affect (SA) and in Restricted and Repetitive Behavior (RRB)^72,73^. CSS can be compared across modules. In our participants, either ADOS G^74^ or ADOS-2^13^ was administered.

For the longitudinal analyses, we computed ADOS and DQ rates of changes using symmetrized percent change (SPC). We used the formula that is displayed in Eq. 1^71,75^.

1 $$\,{SPC}\left[ \% /{year}\right]=100\times \frac{({B}_{y}-{B}_{x})/[({B}_{x}+{B}_{y})/2]}{({{age}}_{y}-{{age}}_{x})}$$

${B}_{x}$ and ${B}_{y}$ are the measures (ADOS CSS or DQ) at ${{age}}_{x}$ and ${{age}}_{y}$, respectively. Each participant’s SPC thus expressed his/her rate of change in either the ADOS or the MSEL as a percentage of his/her mean score across the two timepoints. Furthermore, SPC was normalized for the time difference between the two timepoints, resulting in a yearly rate of change. Using SPC instead of simple absolute difference (such as the raw difference ${B}_{y}-{B}_{x}\,$) to represent longitudinal changes presents many advantages over other measures. These advantages are discussed in Berry & Ayers (2006)^76^ and include decreased sensitivity to outliers, higher statistical robustness, and higher reliability in small samples.

We included participants aged from 1.5 to 6 years old with at least one videotaped ADOS administered by a male examiner (126 recordings across 88 participants). We excluded female examiners to maximize the acoustic difference between child and adult voices and increase the diarization performance. From the 126 recordings, only 10 were collected in typically developing children (TD). We excluded these children because of low sample size, resulting in 116 longitudinal recordings from 78 participants with ASD. Recordings in which total child’s vocalizations did not exceed 30 sec after complete audio preprocessing were excluded (7 recordings from 4 children), resulting in 109 recordings from 74 participants.

To avoid the potential confounding effect of utterances’ linguistic complexity on prosodic measures, we split the sample in two groups, each with a relatively homogeneous level of language. We separated participants according to speech production status defined by the ADOS module they were administered, i.e., either with Phrase Speech (Sp, Modules 2 and 3) or without (PreSp, Module 1 and Toddler). Only one recording per participant was included by applying following inclusion criteria: first, we prioritized Module 2 and 3 (i.e., with Phrase Speech) when available as they were less administered (37 recordings) than PrSp modules (72 recordings) to build more balanced groups in number. Then, when more than one recording of the same speech acquisition status were available (e.g., one Module 2 and one Module 3 for the same participant), we selected the one performed at the youngest age given that we aimed to explore the earliest possible prosody. This resulted in a final sample of 74 participants (32 Sp, aged 4.4 y. ± 0.9, range 2.4–5.9, 4 females, and 42 PreSp aged 3.5 y. ± 1.1, range 2.0–6.5, 7 females). All ADOS were conducted in French and all participants were exposed to French. Detailed sample characteristics can be found in Table 1.

**Table 1:** Sample characteristics with statistical comparison between participants without phrase speech (PreSp) and those with speech (Sp).

MEASURE	Total sample	PreSp	Sp	*P*-Val
Number of participants	74	42	32	
Chronological age [years Mean (SD)]	3.9 (1.1)	3.5 (1.1)	4.4 (0.9)	*p* < 0.001
Gender [females Number (percentage)]	11 (14.9%)	7 (16.7%)	4 (12.5%)	*p* = 0.747 (χ2)
ADOS CSS total [Mean (SD)]	7.4 (1.6)	7.6 (1.7)	7.0 (1.5)	*p* = 0.047 (MW)
ADOS CSS SA	6.0 (1.8)	6.3 (1.9)	5.7 (1.6)	*p* = 0.17 (MW)
ADOS CSS RRB	9.3 (1.0)	9.6 (0.8)	9.0 (1.0)	*p* = 0.006 (MW)
Total DQ [Mean (SD)]	75.0 (27.7)	59.7 (22.8)	95.2 (19.5)	*p* < 0.001
Receptive Language DQ	70.0 (34.3)	51.8 (29.8)	93.8 (23.6)	*p* < 0.001
Expressive Language DQ	63.5 (30.9)	46.3 (24.1)	86.1 (23.5)	*p* < 0.001 (MW)
Visual Reception DQ	85.9 (30.9)	71.9 (26.6)	104.3 (26.4)	*p* < 0.001
Fine Motor DQ	79.9 (22.2)	68.6 (18.8)	94.8 (17.14)	*p* < 0.001
ADOS duration [minutes Mean (SD)]	50.9 (13.6)	42.4 (8.2)	62.1 (10.8)	*p* < 0.001 (MW)
Children vocal production duration [minutes Mean (SD)]	3.0 (2.8)	1.7 (1.4)	4.7 (3.2)	*p* < 0.001 (MW)

Mann–Whitney was used instead of *T*-Tests when normality was not assumed according to Shapiro–Wilk test.

Two recording devices were used: either a Shure© MX202 Microflex® overhead microphone hanging from the ceiling in the middle of the room, or the in-built microphone of the GoPro© Hero7 set in the corner of the room. Analyzing prosody using the ADOS comes with many advantages. Firstly, the ADOS reproduces a naturalistic social interaction, thus eliciting spontaneous prosodic production in a social context. Secondly, compared to home-recordings for instance, the semi-structured setting of the ADOS presents the advantage of greatly reducing variability due to the recording context in terms of physical (e.g., room versus open-air) and social environments (e.g., number of speakers, type of activities engaged, etc.). Finally, the ADOS is the gold standard diagnostic tool used worldwide, making results more generalizable and easily reproducible.

### Filtering and automatic voice detection

The whole preprocessing pipeline of auditory files is summarized in Fig. 4. Firstly, audio tracks from ADOS recordings were extracted with 16-bit and a sampling rate of 48000 Hz. Secondly, voice enhancement was applied to improve voice detection and diarization performance. Voice enhancement comprised a pre-emphasis filter with parameters displayed in Eq. 2^77^ and the spectral subtraction algorithm for speech enhancement implemented in the VOICEBOX toolbox^78–80^.

2 $$P\left(z\right)=1-0.68{z}^{-1}\,$$

Finally, we applied the automatic voice detection algorithm described in Giannakopoulos (2009)^81^. Segments that were not labeled as voice by the algorithm were eliminated from the track. We used Matlab® 2018b for all the preprocessing steps described.

### Speaker diarization algorithm

We built an algorithm trained to classify recorded voice as coming from an adult or a child speaker (i.e., diarization). Importantly, our objective was to get vocal segments classified as belonging to children with minimal misclassification rates, to then alleviate manual editing required to get tracks containing only children’s voices. Thus, correctly classifying audio chunks coming from adult speakers (e.g., to compute adult-child turn-taking) was not the scope of the present method.

To train our classifier, the fundamental frequency (F0) and the first two formants (F1 and F2) were chosen a priori as discriminant acoustic features between child and male adult voices based on the literature^82,83^. For each participant, the audio track that had first undergone voice enhancement and detection was then chunked into 250 ms segments and F0, F1 and F2 were computed within each of those chunks. The F0 was extracted using the in-built Matlab® *F0* function. F1 and F2 were computed for each segment of 5 ms using a linear predictive coding (LPC) approach^84^. When LPC failed to compute the formants (e.g., in unvoiced chunks), values from the previous chunk were used. Moreover, chunks with F1 value superior to 1500 Hz and/or F2 value superior to 6000 Hz were considered as highly improbable^85^ and replaced by the F1 and F2 values of the previous chunk. Then, F1 and F2 values were averaged over chunks of 250 ms to get one F1 and one F2 value for each 250 ms chunk. Finally, values of F0, F1 and F2 of each 250 ms chunk were converted into mel frequencies using the VOICEBOX Toolbox^86^.

To build the diarization algorithm, we first built a training sample consisting of 18 min of voices that had been previously manually classified (9 min of three TD children voices and 9 min of three male adult examiners). To create this training dataset, we randomly selected 3 ADOS recordings of typically developing (TD) children (1 male aged 2.5 y.o. and 2 females aged 3.1 and 3.0) in the database of the *Geneva Autism Cohort* study. In addition, we randomly selected three ADOS recordings performed by three different male examiners. We first applied voice enhancement and automatic voice detection on these tracks. MG then manually extracted 3 min of each child’s voice (9 min in total) and 3 min of each adult male eaminer’s voice (9 min in total) using Audacity® 2.4.2. Segments with overlapping adult and child voice were discarded. Quadratic Fisher Discriminant (QFD) was applied on the training dataset to classify each 250 ms chunk as belonging to either adult or child speaker. Mel frequencies of F0, F1 and F2 were used. Using this approach, the QFD correctly classified 66.0% of the true children chunks and 89.7% of the true adult ones. In general, 79.7% of chunks that were classified as child were true child voice. We enhanced the performance of the classifier by applying the following adjustment: we decided to consider as misclassified all the sequences shorter than 500 ms that had been classified in one condition (child or adult). I.e., a vocalization that lasted <500 ms was not considered as possible. The 500 ms threshold is close to the 600 ms that has been used by Oller et al. (2010)^39^ to define the minimal duration of a human vocalization—this value roughly corresponds to the duration of either two short or one long syllables. The algorithm thus considered as adult voice all sequences shorter than 500 ms that had initially been classified as child voice (and reciprocally). Applying this adjustment, the model correctly classified 70.9% of the true children voice and 98.3% of the true adult ones. In total, 96.1% of the chunks classified as child were true child voice.

We then built a validation sample consisting of 12 min of voices (6 min of children voices from three different TD children and 6 min of three different male adult examiners, extracted manually by MG). Children were not the same as in the training sample (one male aged 3.8 y and two females aged 2.5 y and 2.6 y) whereas adult examiners were the same. After the application of voice enhancement and voice detection on the full audio tracks, MG manually extracted the three adult male examiners and the three TD children voices (2 min of voices for each ADOS, resulting in a total of 6 min of child and 6 min of adult voice). The validation sample was then segmented into 250 ms chunks on which we applied our classifier (that had been first trained on the training sample, and using the adjustment for re-classification of vocalizations shorter than 500 ms). Our classification model showed great generalization performance on the validation dataset. In this sample, 81.6% of the true child voice segments were correctly classified, and 87.5% of segments classified as child voice were true child voice. This means that 12.5% of the data classified as child voices would have to be manually removed before the prosodic analyses (i.e., true adult voice misclassified as child voice), and that 18.5% of the child voice data would be lost for the analyses (i.e., true child voice misclassified as adult voices). Performances of the classifier on the validation sample are displayed on Supplementary Fig. 1.

Here, we limited our sample to ADOS recorded with a single male adult examiner. This restriction aimed at optimizing the diarization algorithm performance as a first approach. Nevertheless, we also explored how our diarization method could apply on recordings of a child interacting with a female adult. F0, F1 and F2 also exhibit differences between female adults and children and could thus be used to diarize these populations. Nonetheless, lower accuracy is expected given that these parameters in adult females are less different from children compared to adult males, making discrimination more difficult. We explored three different approaches.

First, we applied the classifier that had been first trained to classify adult male/children voices on a validation sample that consisted in child and female adult voices. The aim was to estimate how well the initial classifier generalized to female adult voice. We randomly selected three ADOS performed by three different adult women and MG manually extracted their voices (2 min for each examiner, 6 min in total). The same three TD child participants as the ones used in the validation sample described above were used. Using this approach, 81.6% of the true child voice and 32.6% of the true adult female voice were correctly classified. The chunks classified as child were correct 40.6% of the time. This means that 59.4% of the resulting audio track would have to be manually removed before analysis when using this approach. Moreover, 81.6% of the true children voices were correctly identified, meaning that there was no data loss compared to the same classifier applied on recordings with adult males.

As a second approach, we trained a whole new classifier on a sample consisting of children and adult female examiners voice (i.e., using female voices in both the training and the validation datasets). To build the training sample, we randomly chose three ADOS recordings performed by three different female examiners and MG extracted 3 min of each examiners’ voice (9 min in total). QFD was trained on these voices using the same three children participants as the ones used in the training sample for the adult male/child classifier (9 min of children voice). On this training sample, 40.5% of the true child voice and 91.3% of the true adult female voice were correctly classified. The chunks classified as child were correct 73.6% of the time. The resulting classifier was then applied on the validation sample with children voices and adult female voices in which 11.6% of children voices were correctly classified (meaning that 88.4% of the true child voice data would be lost) and the chunks classified as child were correct 41.0% of the time.

Finally, we trained a three-way classifier using all three voice categories (children, adult female, and adult male). We mixed the children, adult female, and male true voices of all training samples (9 min of isolated voices for each voice category) and trained the three-way QFD to classify them. On the training sample, 87.5% of the true adult female voice, 76.9% of the true adult male voice and 27.6% of the true children voice were correctly identified. The validation sample then consisted in the 6 min of each voice category that we had already used in the approaches described above. Performances of the three-way classifier on the validation sample are displayed on Supplementary Fig. 2. The model correctly identified 64.7% of the true adult male voice, 78.5% of the true adult female voice and 44.7% of the true children voice. Altogether, 44.7% of the true children voices were correctly identified (i.e., 55.3% of true child voice data loss), and the resulting track contained 30.8% of incorrectly classified true adult voice data that would have to be manually removed.

### Manual editing

MG carefully inspected all the segments classified as child voice by the diarization algorithm. Audacity® 2.4.2 (Audacity® software is copyright © 1999–2021 Audacity Team, the name Audacity® is a registered trademark) was used to remove all segments mistaken for a child voice but being either adult voice or background noise that had not been removed by the voice detection algorithm. Segments consisting of child’s voice overlapping with background noise or the examiner’s voice were also removed.

### Prosodic parameters

We used the Geneva Minimalistic Acoustic Parameter Set (GeMAPS)^41^ to extract acoustic features related to prosody. GeMAPS was run via audEERING® opensmile v2.4.2^87^. GeMAPS automatically quantifies a set of prosodic parameters and has already been used in ASD^88,89^. For each participant, GeMAPS automatically chunks the audio recording and computes various acoustic parameters (e.g., pitch) within each chunk. Then, it gives statistics for each parameter (e.g., mean and standard deviation of pitch over all chunks for a given participant). Here, we extracted the following parameters related to intonation from the GeMAPS:

- **Pitch** (semi-tone scale starting at 27.5 Hz)
- **Pitch variability**
- **Rising Pitch** (Mean of the slope of the rising pitch segments)
- **Rising Pitch Variability**
- **Falling Pitch**
- **Falling Pitch Variability**

To this GeMAPS set of intonation-related parameters we added the *pitch excursion*. We used the formula introduced by De Pijper (1983)^90^ as this metric has been found to be increased in ASD^21^. Briefly, within each chunk (1 s duration) we computed the logarithm of the maximal F0 divided by the minimal F0, multiplied by 39.863 and divided by the chunk duration. This gives a value in semitone/second that captures the magnitude of local intonation changes.

We computed the following parameters related to loudness:

- **Rising Loudness** (Mean of the slope of loudness of the segments with rising loudness)
- **Rising Loudness Variability**
- **Falling Loudness**
- **Falling Loudness Variability**

As the distance between the child and the microphone was not kept constant during recording (stationary microphone and child freely moving in the room), we did not use the mean loudness parameter as it would not be standardized between children. In contrast, rising and falling loudness parameters are based on the loudness slope within very short chunks. We can approximate that the child is static within one chunk so that the inconsistent distance between the child and the microphone is less affecting these two dynamical loudness parameters.

We used the following parameters related to voice quality:

- **Jitter** (variability in the length of the consecutive F0 period lengths)
- **Jitter variability**
- **Shimmer** (variability in the amplitude of consecutive F0 periods)
- **Shimmer variability**
- **Harmonic to Noise Ratio (HNR)** (Energy of harmonic components divided by noise-like ones)
- **H1-H2** (Energy of the first F0 harmonic divided by the second one)
- **H1-H2 variability**
- **H1-A3** (Energy of the first F0 harmonic divided by the energy of the highest harmonic found in the range of F3)
- **H1-A3 variability**
- **F1, F2 and F3** (Three first formants)
- **F1, F2 and F3 variability**

We used the following parameters associated to rhythm:

- **Pseudo Syllable Rate** (number of voiced segments per second)
- **Vowel length**
- **Vowel length variability**
- **Loudness peaks/second**

### Partial least square correlations

We explored the multivariate relations between behavior and prosody by applying partial least square correlations (PLSC)^91,92^. We used the myPLS toolbox^93^ with Matlab® 2018b. The toolbox is publicly available (https://github.com/danizoeller/myPLS). PLSC first computes a correlation matrix between prosodic features and behavior measures. In PLSC, behavior measures can consist of either continuous measures (e.g., DQs) or discrete contrasts (e.g., group belonging). The correlation matrix is then decomposed into singular values and several correlation components are derived from singular values. Each of these correlation components results from the combination of behavior and prosodic saliences, that reflect how much each variable contributes to the multivariate behavior-prosody correlation component. We used permutation testing to determine the statistical significance of each correlation component (1000 permutations). We only considered significant correlation components with *p* < 0.05 after permutation testing. Then, we used bootstrapping with 500 bootstrap samples and replacement to determine the stability of each behavior and prosody variable saliency. Saliencies were considered stable when their bootstrap ratio (BSR, mean divided by standard deviation of saliency bootstrapping result) were >2.3, corresponding to a confidence interval of 99% that does not cross zero, thus indicating a stable contribution of the measure to the correlation component^91^. Age, gender, and the type of microphone used were regressed out as nuisance factors. When PLSC was applied using a behavioral discrete contrast (when comparing prosody between PreSp and Sp), nuisance factors were regressed out within each group separately, whereas normalization, bootstrapping and permutation were performed across all subjects. We kindly refer the reader to Zöller et al. (2019) for a more detailed description of the PLSC algorithm and parameters we applied^94^.

We first measured how prosody relates to behavior within each group separately (PreSp and Sp). We thus ran a PLSC on the PreSp sample (*n* = 42) and a second one on the Sp sample (*n* = 32) using autism symptom severity (measured with ADOS CSS) and developmental level (measured with DQ) as behavioral variables.

We then explored the effect of potential confounding factors on prosodic metrics, namely linguistic complexity of utterances and age. We defined linguistic complexity according to the speech acquisition status and we ran PLSC on the whole sample (*n* = 74) using group belonging (PreSp or Sp) as the single behavioral contrast. Furthermore, we ran a PLSC on the whole sample using age as the single behavioral variable.

Finally, we examined how prosody in preschoolers without phrase speech (PreSp) predicted behavioral rates of change in the subsequent year. We isolated a subgroup within the PreSp group for which ADOS and DQ had been collected 1 year later (*n* = 32, aged 3.3 y.o. ± 0.9, range 3.3–5.2, 7 females, see Table 2). Yearly rates of change were computed using the symmetrized percent change (SPC) described above^76^. We ran PLSC on this longitudinal subsample using prosody metrics and SPC values for DQ and ADOS CSS.

**Table 2:** Demographic and behavioral measures in the subsample of PreSp participants with ADOS and DQ collected 1 year after the recording (*n* = 32).

MEASURE	PreSp longitudinal sample
Number of participants	32
Chronological age [years Mean (SD)]	3.3 (0.9)
Gender [females Number (percentage)]	7 (22%)
ADOS CSS total [Mean (SD)]	7.7 (1.8)
ADOS CSS SA	6.3 (2.1)
ADOS CSS RRB	9.7 (0.8)
Composite DQ [Mean (SD)]	56.2 (21.6)
Fine Motor DQ	64.6 (19.8)
Visual Reception DQ	67.0 (24.3)
Expressive Language DQ	44.2 (22.8)
Receptive Language DQ	49.0 (28.7)
Composite DQ SPC [%/yr Mean (SD)]	10.7 (25.4)
Fine Motricity DQ SPC [%/yr Mean (SD)]	5.3 (28.1)
Visual Reception DQ SPC [%/yr Mean (SD)]	11.0 (30.1)
Expressive Language DQ SPC [%/yr Mean (SD)]	19.2 (32.6)
Receptive Language DQ SPC [%/yr Mean (SD)]	6.9 (39.3)
ADOS CSS total SPC [%/yr Mean (SD)]	-1.0 (24.2)
ADOS CSS SA SPC [%/yr Mean (SD)]	2.7 (37.2)
ADOS CSS RRB SPC [%/yr Mean (SD)]	-0.6 (12.5)
ADOS duration [minutes Mean (SD)]	43.0 (8.9)
Children vocalizations duration [minutes Mean (SD)]	1.9 (1.6)

SPC relates to symmetrized percent change, a measure of rate of change within the subsequent year (see supplementary material).

Age, gender, and the type of microphone used were regressed out as nuisance factors in all analyses.

### Reporting summary

Further information on research design is available in the Nature Research Reporting Summary linked to this article.

## Supplementary information

Reporting Summary

Supplementary Material

## Data Availability

Raw behavioral and prosodic measures used to support the conclusion of this study will be made available by the corresponding author on reasonable request. Video and audio recordings from participants represent sensitive data that allow a potential personal identification and thus cannot be shared.

## Code availability

The Matlab® code developed for diarization algorithm we present here will be made available by the corresponding author on reasonable request.
