# Supplementary material for: Prosodic signatures of ASD severity and developmental delay in preschoolers
Source: NPJ Digit Med. 2023 May 29;6:99. doi: 10.1038/s41746-023-00845-4 (PMC10227036; doi:10.1038/s41746-023-00845-4)
Supplement: Supplementary file 1 — Reporting Summary [file 41746_2023_845_MOESM1_ESM.pdf]

nature portfolio

Corresponding author(s): NPJDIGITALMED-05622R

Last updated by author(s): Feb 24, 2023

Reporting Summary

Nature Portfolio wishes to improve the reproducibility of the work that we publish. This form provides structure for consistency and transparency in reporting. For further information on Nature Portfolio policies, see our Editorial Policies and the Editorial Policy Checklist.

Statistics

For all statistical analyses, confirm that the following items are present in the figure legend, table legend, main text, or Methods section.

n/a	Confirmed	
☐	☒	The exact sample size (*n*) for each experimental group/condition, given as a discrete number and unit of measurement
☐	☒	A statement on whether measurements were taken from distinct samples or whether the same sample was measured repeatedly
☐	☒	The statistical test(s) used AND whether they are one- or two-sided<br>*Only common tests should be described solely by name; describe more complex techniques in the Methods section.*
☐	☒	A description of all covariates tested
☐	☒	A description of any assumptions or corrections, such as tests of normality and adjustment for multiple comparisons
☐	☒	A full description of the statistical parameters including central tendency (e.g. means) or other basic estimates (e.g. regression coefficient) AND variation (e.g. standard deviation) or associated estimates of uncertainty (e.g. confidence intervals)
☐	☒	For null hypothesis testing, the test statistic (e.g. *F*, *t*, *r*) with confidence intervals, effect sizes, degrees of freedom and *P* value noted<br>*Give P values as exact values whenever suitable.*
☐	☒	For Bayesian analysis, information on the choice of priors and Markov chain Monte Carlo settings
☐	☒	For hierarchical and complex designs, identification of the appropriate level for tests and full reporting of outcomes
☐	☒	Estimates of effect sizes (e.g. Cohen's *d*, Pearson's *r*), indicating how they were calculated

Our web collection on statistics for biologists contains articles on many of the points above.

Software and code

Policy information about availability of computer code

Data collection	No software was used
Data analysis	Audacity® 2.4.2 was used for auditory manual editing. Matlab® 2018b was used for all statistical analyses and auditory preprocessing. Open source VOICEBOX Toolbox was used for spectral substraction. Matlab detectSpeech function was used for voice detection. Publicly available GeMAPS provided by Opensmile v2.4.2 (AudEERING®) was used to extract prosodic measures. Open source toolbox "myPLS toolbox" was used for multivariate analyses.

For manuscripts utilizing custom algorithms or software that are central to the research but not yet described in published literature, software must be made available to editors and reviewers. We strongly encourage code deposition in a community repository (e.g. GitHub). See the Nature Portfolio guidelines for submitting code & software for further information.

Data

Policy information about availability of data

All manuscripts must include a data availability statement. This statement should provide the following information, where applicable:

- Accession codes, unique identifiers, or web links for publicly available datasets
- A description of any restrictions on data availability
- For clinical datasets or third party data, please ensure that the statement adheres to our policy

Raw behavioral and prosodic measures used to support the conclusion of this study as well as the Matlab® code developed for diarization will be made available by

the corresponding author on reasonable request. Video and audio recordings from participants represent sensitive data that allow a potential personal identification and thus cannot be shared.

Human research participants

Policy information about studies involving human research participants and Sex and Gender in Research.

Reporting on sex and gender	Findings apply to both genders as this information has been regressed out from analyses as a nuisance factor.
Population characteristics	All participants had an ASD diagnosis confirmed by a licensed child psychiatrist using the Diagnostic and Statistical Manual of mental disorders, 5th edition criteria, combined with the Autism Diagnostic Observation Schedule (ADOS) diagnostic cut-off. Total sample (n=74) characteristics: Mean age 3.9 years old, 14.9% of female participants, Mean ASD symptom severity (calibrated severity score CSS) 7.4, Mean Developmental Quotient 75.0,
Recruitment	Preschoolers with either typical development (TD) or Autism Spectrum Disorder (ASD) were recruited through parent associations, clinical centers and announcements in the Geneva community. ASD diagnosis was confirmed by a licensed child psychiatrist using the Diagnostic and Statistical Manual of mental disorders, 5th edition criteria, combined with the Autism Diagnostic Observation Schedule (ADOS) diagnostic cut-off.
Ethics oversight	The research protocol was approved by the review board of the University of Geneva

Note that full information on the approval of the study protocol must also be provided in the manuscript.

Field-specific reporting

Please select the one below that is the best fit for your research. If you are not sure, read the appropriate sections before making your selection.

☒ Life sciences ☐ Behavioural & social sciences ☐ Ecological, evolutionary & environmental sciences

For a reference copy of the document with all sections, see nature.com/documents/nr-reporting-summary-flat.pdf

Life sciences study design

All studies must disclose on these points even when the disclosure is negative.

Sample size	We applied Partial Least Square Correlation, a multivariate approach that is suited for small sample sizes. There is no consensus about how to predetermine minimal sample size for a given power with this approach.
Data exclusions	We excluded recordings comprising female examiners to maximize the acoustic difference between child and adult voices, and increase the diarization performance. We excluded typically developing children because of low sample size (n=10). . Recordings in which total child's vocalization did not exceed 30 seconds after complete audio preprocessing were excluded (4 participants). Only one auditory recording per participant was included: the one performed at youngest age with speech was selected. If no recording with speech was available for one participant, recording at youngest age was selected.
Replication	No replication of the results presented here was attempted in the present study. Measures used in the behavioral domain (MSEL, PEP-3 and ADOS 2) are widely used in ASD research and show high rates of reproducibility. Measures used in prosodic domain (GeMAPS features) are commonly used in prosodic research and are publicly available.
Randomization	Not relevant here as this is an observational study (we do not compare a treatment/task between groups)
Blinding	Main investigator was blind to the participants clinical characteristics (age, gender, developmental level and autism symptom severity) when manually preprocessing the voices.

Reporting for specific materials, systems and methods

We require information from authors about some types of materials, experimental systems and methods used in many studies. Here, indicate whether each material, system or method listed is relevant to your study. If you are not sure if a list item applies to your research, read the appropriate section before selecting a response.

Materials & experimental systems

n/a	Involved in the study
☒	☐ Antibodies
☒	☐ Eukaryotic cell lines
☒	☐ Palaeontology and archaeology
☒	☐ Animals and other organisms
☒	☐ Clinical data
☒	☐ Dual use research of concern

Methods

n/a	Involved in the study
☒	☐ ChIP-seq
☒	☐ Flow cytometry
☒	☐ MRI-based neuroimaging
