# Supplementary material for: Prosodic signatures of ASD severity and developmental delay in preschoolers
Source: NPJ Digit Med. 2023 May 29;6:99. doi: 10.1038/s41746-023-00845-4 (PMC10227036; doi:10.1038/s41746-023-00845-4)
Supplement: Supplementary file 2 — Supplementary Material [file 41746_2023_845_MOESM2_ESM.pdf]

Supplementary Material

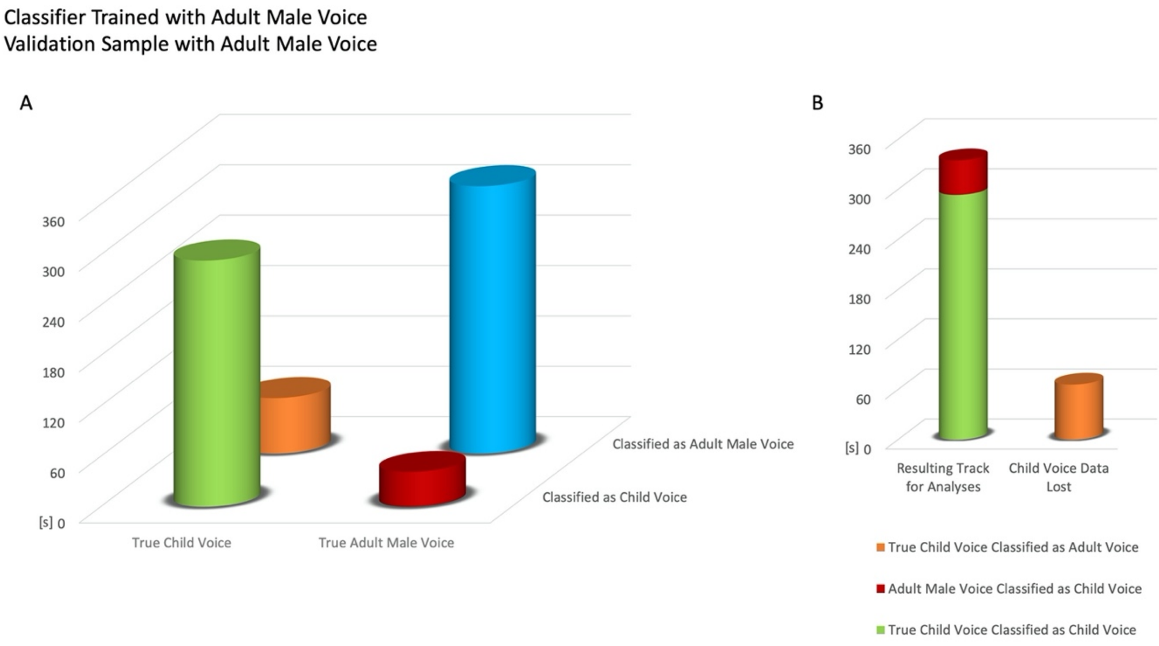

Supplementary Figure 1: A. *Three-dimensional confusion matrix of the classifier that had been first trained on adult male and children voices (9min of each type) and was then applied on a validation dataset comprising 360 seconds (6 min) of adult male voices and 360 seconds of children voices. Classifier achieved 81.6% sensitivity in detecting child voices and 88.5% for adult male voices.* ***B.*** *Data from the panel A that would still have to be manually cleaned before analyses (the "Resulting Track for Analyses"). In green are the true child voice samples that were correctly identified, in red the adult male voice that were incorrectly classified as child voice (i.e., that must be manually removed), and in orange the child voice data that was not identified as child voice and would thus be lost for the analyses.*

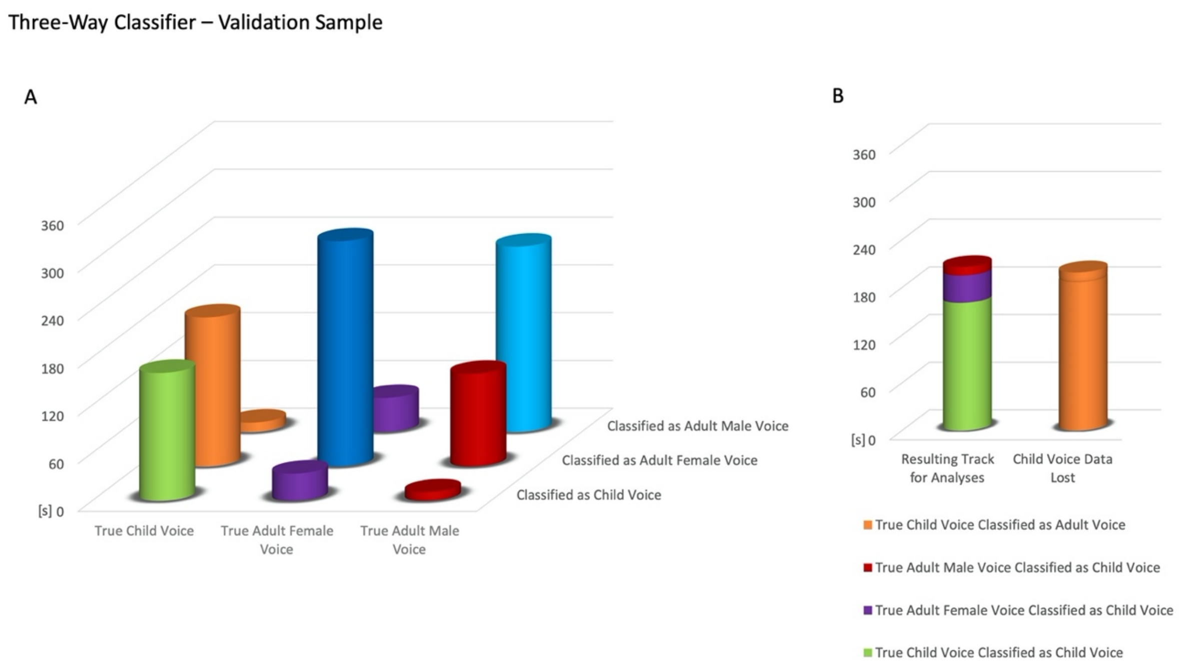

Supplementary Figure 2: A. *Three-dimensional confusion matrix of a three-way classifier that had first been trained to identify three categories of voice: adult male, adult female and children. Here are the performances of the trained classifier when applied on a validation sample comprising all three categories of voice.* ***B.*** *Amount of true child voice data (in green) that was kept for analyses. Amount of adult voice data that would have to be removed manually (incorrectly classified adult male voice in red and female adult in purple). Amount of true child voice data that was lost for the analyses in orange (lower part of the cylinder represents data incorrectly classified as 'adult female' and upper part represents data incorrectly classified as 'adult male').*

Supplementary Table

	PreSp	Sp	Sp > PreSp	PreSp Longitudinal
Social Affect CSS	**4,258**	0,771		
Repetitive and Restricted Behavior CSS	**4,260**	**2,353**		
Receptive Language	**-6,717**	**-10,389**		
Expressive Language	**-5,660**	**-5,861**		
Visual Reception	**-5,257**	**-7,178**		
Fine Motor	**-8,431**	**-3,361**		
Sp > PreSp			∞	
Social Affect CSS SPC				**3,674**
Repetitive and Restricted Behavior CSS SPC				**-5,429**
Receptive Language SPC				**11,373**
Expressive Language SPC				1,148
Visual Reception SPC				**5,185**
Fine Motor SPC				**4,421**
Pitch	2,276	1,010	**-9,556**	0,226
Pitch variability	-0,937	-0,171	-0,074	**4,245**
Pitch Excursion	**-6,227**	0,754	**4,187**	-0,413
Rising Pitch	-0,555	-0,289	0,528	-1,212
Rising Pitch variability	-0,419	-0,222	1,706	-0,899
Falling Pitch	-0,027	1,606	-0,034	-1,098
Falling Pitch Variability	1,085	0,617	1,321	-1,475
Rising Loudness	-1,356	0,166	-1,235	1,535
Rising Loudness Variability	-0,538	-0,134	-1,730	0,611
Falling Loudness	-0,599	-0,089	-1,105	1,781
Falling Loudness Variability	0,297	-0,476	-2,269	1,219
Jitter	**-2,478**	**-12,139**	-0,031	**2,715**
Jitter Variability	**5,798**	**9,852**	2,211	**7,729**
Shimmer	**-7,758**	**-17,494**	-0,265	**-7,092**
Shimmer Variability	**5,725**	**13,072**	0,001	**10,354**

Harmonic to Noise Ratio (HNR)	**4,405**	0,845	-0,590	0,729
HNR Variability	**-5,994**	**-17,016**	-1,001	**-6,449**
H1-H2	0,774	-1,458	**4,223**	1,513
H1-H2 Variability	0,366	-2,582	**-2,895**	-1,378
H1-A3	-1,072	-0,813	**2,955**	**2,483**
H1-A3 Variability	1,310	-2,463	-1,455	**2,624**
F1	1,450	1,284	**-11,000**	0,774
F1 Variability	1,073	**-2,966**	0,161	**5,529**
F2	1,367	-1,154	**-2,434**	0,690
F2 Variability	2,183	**3,890**	0,153	**3,374**
F3	-0,010	-0,554	-1,669	1,708
F3 Variability	1,475	-2,260	0,138	**-6,293**
Pseudo Syllable Rate	**-3,717**	**7,251**	**3,598**	**-4,044**
Vowel length	1,933	**-15,361**	-2,213	**4,320**
Vowel length Variability	**5,281**	**-13,660**	**-2,656**	**4,142**
Loudness Peaks / s	**-2,817**	-2,187	1,234	**-2,649**

Supplementary Table: Bootstrap Ratios (BSR) of each variable (rows) for significant correlation components of the Partial Least Square Correlation (PLSC) analyses presented in the Results section. Each column represents the significant correlation component of one PLSC. BSR for the PLSC within PreSp (first column), within Sp (second column), in the whole sample using language production stage as a binary contrast (third column) and in a subset of PreSp with longitudinal data (fourth column). Above the horizontal double line are the behavioral variables, beneath are the prosodic variables. BSR > 2.3 are highlighted in bold (BSR considered as "stable"). CSS: Calibrated Severity Score; SPC: Symmetrized Percent Change.
